# Overexpression of angiopoietin 2 promotes the formation of oral squamous cell carcinoma by increasing epithelial–mesenchymal transition-induced angiogenesis

**DOI:** 10.1038/cgt.2016.30

**Published:** 2016-08-05

**Authors:** C Li, Q Li, Y Cai, Y He, X Lan, W Wang, J Liu, S Wang, G Zhu, J Fan, Y Zhou, R Sun

**Affiliations:** 1 Department of Head and Neck Surgery, Sichuan Cancer Hospital, Chengdu, China; 2 Southwest Medical College, Luzhou, China; 3 The Fifth People's Hospital of Chengdu, Chengdu, China; 4 Chengdu Medical College, Chengdu, China

## Abstract

Oral squamous cell carcinoma (OSCC) is the most common cancer of the head and neck and is associated with a high rate of lymph node metastasis. The initial step in the metastasis and transition of tumors is epithelial–mesenchymal transition (EMT)-induced angiogenesis, which can be mediated by angiopoietin 2 (ANG2), a key regulatory factor in angiogenesis. In the present study, immunohistochemistry and real-time quantitative reverse transcriptase (qRT-PCR) were used to measure the expression of ANG2 in OSCC tissues. Plasmids encoding ANG2 mRNA were used for increased ANG2 expression in the OSCC cell line TCA8113. The short interfering RNA (siRNA)-targeting ANG2 mRNA sequences were used to inhibit ANG2 expression in TCA8113 cells. Subsequently, transwell assays were performed to examine the effects of ANG2 on TCA8113 cell migration and invasion. Furthermore, *in vivo* assays were performed to assess the effect of ANG2 on tumor growth. Terminal deoxynucleotidyl transferase dUTP nick-end labeling (TUNEL) assays and immunohistochemistry were used to examine cell apoptosis and angiogenesis in tumor tissues, respectively. Finally, western blot analysis was performed to evaluate tumor formation-related proteins in OSCC tissues. We found that protein expression of ANG2 was remarkably upregulated in OSCC tissues. Overexpression of ANG2 increased the migration and invasion of TCA8113 cells by regulating EMT. Further investigations showed that overexpression of ANG2 increased tumor growth in nude mice, and angiogenesis of OSCC tissues increased in the presence of ANG2 overexpression. Overexpression of ANG2 also reduced cell apoptosis in tumor tissue cells. Finally, we found that overexpression of ANG2 resulted in changes in the expression of tumor formation-related proteins including vimentin, E-cadherin, Bim, PUMA, Bcl-2, Bax, Cyclin D1, PCNA and CD31. Our findings show that ANG2 has an important role in the migration and invasion of OSCC. More importantly, further investigations suggested that overexpression of ANG2 might increase OSCC metastasis by promoting angiogenesis in nude mice. This stimulatory effect could be achieved by inducing abnormal EMT and by reducing apoptosis and increasing proliferation of cells.

## Introduction

Oral squamous cell carcinoma (OSCC), the most common cancer of the head and neck, is associated with a high rate of lymph node metastasis and a poor prognosis.^1^ Although progress has been achieved in the treatment of OSCC by surgery, chemotherapy and radiotherapy, the clinical applications are limited by efficacy and toxicity. The migration and invasion of cancer cells are considered to be important characteristics of systemic cancer morbidity and mortality. Therefore, it is important for us to understand the molecular bases of metastasis in OSCC to decrease the mortality rate and to determine the patient prognosis.

Tumor metastasis requires that tumor cells acquire invasion activity by activating an epithelial–mesenchymal transition (EMT) program to undergo phenotypic changes such as the loss of cell–cell adhesion and the gain of cell migration capabilities to evade the primary tumor.^2^ Angiogenesis is the process of the formation of new and irregular blood vessels from the pre-existing vasculature.^3^ EMT-induced abnormal angiogenesis is the fundamental step in the transition of tumors from a primary state to a malignant one, a process that is regulated by many bioactive molecules.^3, 4^

Angiopoietin 2 (ANG2), a member of the angiopoietins, is thought to have an important role in angiogenesis.^5^ A previous study showed that increased ANG2 can result in persistent disruption of the cellular crosstalk between endothelial cells and pericytes.^6^ Furthermore, high concentrations of ANG2 are considered to function as anti-apoptotic factors in endothelial cells by activating the phosphatidylinositol 3′-kinase/Akt signaling pathway.^7^ In addition, ANG2 expression is significantly upregulated in poorly differentiated hepatocellular carcinoma.^8^ Additional studies have shown that ANG2 overexpression is associated with lymph node metastasis,^9^ and high ANG2 expression is significantly associated with advanced-stage tumors, a positive nodal stage and distant metastasis in gastric cancer.^10^ These reports suggest that tumor aggression may be the result of the promotion of angiogenesis regulated by ANG2. However, the role of ANG2 in OSCC development is not well known, and the detailed mechanism of ANG2-mediated angiogenesis promoting tumor progression is still debated.

In the present study, we found that ANG2 was remarkably upregulated in OSCC tissues, and OSCC tissue progression was tightly linked to ANG2-mediated angiogenesis. Further investigations explored the molecular basis of ANG2 overexpression in the promotion of tumor formation. These data provide new insights into the role of ANG2 during the process of OSCC.

## Materials and methods

### Cells and transfection

The human OSCC cell lines TCA8113, cal27, SCC4, SCC15 and SCC25 were obtained from the China Center for Type Culture Collection (Wuhan, China). These cells were grown in RPMI-1640 medium (GIBCO, Scotland, UK), supplemented with 10% fetal bovine serum (GIBCO) at 37 °C and 5% CO_2_ in a humidified atmosphere. For transfection, cells in the monolayer were transfected with the plasmid encoding ANG2 mRNA and ANG2-targeted short interfering RNA (siRNA; 5′-GGACAAACCUGUUGAACCAAA-3′) using FuGENE HD transfection reagent (Roche, Basel, Switzerland) according to the manufacturer's protocol. All subsequent assays were performed 24 h after transfection, except where indicated otherwise.

### Immunohistochemistry

Three pairs of paraffin-embedded tissue blocks were collected from OSCC and normal tissues. Every block was cut into 4-mm slices using a microtome and placed on a slide. The tissue sections were dewaxed in xylene and rehydrated through graded alcohol concentrations according to standard procedures. The sections were subsequently submerged in EDTA (pH 8.0) and autoclaved at 121 °C for 5 min for antigen retrieval. After washing three times in phosphate-buffered saline (PBS), endogenous peroxidase activity was neutralized by the addition of 3% (v/v) H_2_O_2_ followed by incubation for 15 min at room temperature. After washing with PBS, the sections were blocked with 3% bovine serum albumin at room temperature for 5 min, followed by an overnight incubation at 4 °C with ANG2 (Santa Cruz Biotechnology, Santa Cruz, CA, USA, sc-74402) or CD31 (Santa Cruz Biotechnology, sc-7382) antibody (diluted 1:100 in PBS, 0.5% bovine serum albumin). The sections were washed and incubated with biotin-labeled antibody, followed by peroxidase-conjugated streptavidin for another 30 min. The three, 30-diaminobenzidine tetrahydrochloride (Dako, Glostrup Kommune, Denmark) was added to visualize the reaction, followed by counterstaining with commercial hematoxylin, followed by sequential dehydration in alcohol and xylene and mounting. Negative PBS controls were obtained by omitting the primary antibodies.

The expression of ANG2 was evaluated by Imageproplus (version 6.0; Media Cybernetics, Silver Spring, MD, USA). Ten digital images with a pixel resolution of × 1360 were captured for every section at × 400 magnification using a BX51WI microscope (Olympus, Tokyo, Japan). The measurement parameters included the density mean, area sum and integral optical density. The optical density was calibrated, the image was converted to gray scale and the values were counted.

### RNA isolation and real-time RT-PCR

Total RNA was extracted from cells using TRIzol (Invitrogen, Carlsbad, CA, USA) according to the manufacturer's protocol. Real-time quantitative reverse transcriptase (qRT-PCR) assays were performed to evaluate the expression of ANG2, and primers were designed as follows: ANG2-Fwd: 5′-TCCAAGAGCTCGGTTGCTAT-3′ ANG2-Rev: 5′-AGTTGGGGAAGGTCAGTGTG-3′ GAPDH-Fwd: 5′-GAGTCAACGGATTTGGTCGT-3′, GAPDH-Rev: 5′-GACAAGCTTCCCGTTCTCAG-3. The ANG2 mRNA expression level was standardized to GAPDH mRNA by the following calculation: ΔCt=Ct (RFC3)−Ct (GAPDH). All experiments were performed at least three times.

### MTS assay

Cell viability was assessed by the MTS (3-(4, 5-dimethylthiazol-2-yl)-5-(3-carboxymethoxyphenyl)-2-(4-sulfophenyl)-2H-tetrazolium) assay. Cells were collected in the logarithmic growth phase and plated at a density of 1 × 10^3^ per well in 96-well plates in triplicate. Following treatment, every well was incubated with MTS reagent in complete medium at a ratio of 1:10 for 4 h. The absorbance value of each well was measured using a microplate reader (Diatek, Wuxi, China) at a wavelength of 490 nm.

### Flow cytometry assay

Cell apoptosis was measured using a fluorescein isothiocyanate Annexin V apoptosis detection kit (BD Pharmingen, Franklin, NJ, USA, 556547) according to the manufacturer's instructions. Briefly, the cells were seeded in six-well plates at ~1 × 10^5^ cells per ml. After a 24-h incubation, the cells were digested, harvested and washed twice in PBS, followed by incubation with Annexin V-fluorescein isothiocyanate and propidium iodide for 15 min in the dark. For cell cycle analysis, the cells were washed with ice-cold PBS and fixed with 70% ethanol overnight at −20 °C. The fixed cells were rehydrated in PBS for 10 min and subjected to propidium iodide/RNase staining. The apoptotic levels and cell cycle in each sample were determined using a FACSCalibur flow cytometer (BD Biosciences, Franklin Lakes, NJ, USA) according to the manufacturer's guidelines.

### Transwell migration and invasion assay

Transwell migration assays were performed using 8-μm-pore transwell chambers in 24-well plates (Corning Costar, Cambridge, MA, USA). After overnight starvation in serum-free RPMI-1640, the cell suspension (4 × 10^5^ cells per ml, 100 μl) was added to the upper chamber. The lower chamber was filled with 800 μl of RPMI-1640 medium containing 10% fetal bovine serum. Subsequently, the cells were cultured for another 12 h. After swabbing the non-migrated cells from the upper chambers, the cells that had migrated to the lower chambers were fixed with 4% paraformaldehyde in PBS and stained with Giemsa. Finally, the cells that had migrated to the lower chambers were observed under a light microscope (× 200). The cell invasion assay procedure was similar to that used for cell migration, except that the transwell membranes were pre-coated with 50 μg μl^−1^ Matrigel (BD Biosciences) and the cells were incubated for 48 h. Cells that had migrated to the lower chambers were quantified as described for the cell migration assay.

### Western blot analysis

Cells or tissues were harvested and lysed in lysis buffer (50 mm Tris, 150 mm NaCl, 1% Triton X-100 and 0.5% deoxycholate) containing 0.5 mm sodium orthovanadate, 1 mm phenylmethylsulfonyl fluoride, 1 mg ml^−1^ aprotinin and 2 μg ml^−1^ leupeptin. The lysates were cleared by centrifugation (14 000 r.p.m.) at 4 °C for 30 min. The protein concentration in the supernatant was determined using a Micro BCA Protein Assay Reagent Kit (Pierce, Rockford, IL, USA). Normalized lysates were boiled in electrophoresis sodium dodecyl sulfate (SDS) sample buffer, run on a 10% SDS-polyacrylamide gel electrophoresis gel and transferred to a polyvinylidine difluoride membrane (Millipore, Boston, MA, USA). The membranes were blocked for 1 h in Tris-buffered saline with 0.05% Tween 20 (TBST) containing 5% skim milk. The membrane was probed with antibodies against vimentin (Santa Cruz Biotechnology, sc-3232), Snail (Abcam, Cambridge, England, ab180714), Twist (Abcam, ab175430), E-cadherin (Abcam, ab1416), Bim (Abcam, 1C2H4), PUMA (Sigma, St. Louis, MO, USA, P4743), Bcl-2 (Abcam, ab692), Cyclin D1 (Santa Cruz Biotechnology, sc-20044), PCNA (Santa Cruz Biotechnology, sc-25280) and GAPDH (Transgene, Graffenstaden, France, HC301), with the proper concentrations and times according to the manufacturer's guidelines. The membrane was then washed twice with TBST for 10 min and incubated with peroxidase-conjugated goat anti-mouse IgG antibody (1:5000; Amersham Pharmacia Biotech, Pittsburgh, PA, USA) for 1 h. After three washes, the membrane was developed using an enhanced chemiluminescence system (Amersham Pharmacia Biotech).

### *In vivo* metastasis assay

BALB/c nude mice were obtained from the Vital River Laboratories, Charles River, Beijing, China, and randomly divided into four groups (*n*=6 in each group, which was suitable for statistical calculations^11^). TCA8113 cells (2 × 10^5^ per 50 μl) transfected with ANG2-encoding plasmid or ANG2-targeted siRNA were injected subcutaneously into the backs of 5-week-old BALB/c nude mice. The tumors were surgically removed and measured with calipers, and the volume was calculated as follows: (length × width^2^) × 0.5 every week. The tumor tissues at 4 weeks were used for the subsequent terminal deoxynucleotidyl transferase dUTP nick-end labeling (TUNEL) assays, western blot analysis and CD31 immunohistochemistry (IHC). All experimental procedures performed in mice were approved by the Institutional Animal Care and Use Committee of Sichuan Cancer Hospital, Chengdu, China.

### TUNEL assays

Cells containing fragmented DNA were evaluated in paraffin sections using a fluorometric TUNEL kit (Promega, Madison, WI, USA, G3250) according to the manufacturer's protocol. Briefly, the sections were dewaxed in xylene and rehydrated through a decreasing series of ethanol concentrations in water. The sections were immersed in 0.85% NaCl for 5 min, washed in PBS, fixed in 4% paraformaldehyde in PBS for 15 min and washed twice in PBS. To permeabilize the sections and allow antigen unmasking, the slides were treated with hot (>85 °C) citrate buffer for 10 min and then cooled for 20 min before being washed with PBS. The sections were incubated with 100 μl of equilibration buffer for 10 min and then labeled with 50 μl of TUNEL reaction mix for 1 h at 37 °C. The reactions were stopped by a 15-min wash in × 2 saline-sodium citrate buffer. The sections were washed in PBS to remove excess label and then dyed with 5 μg ml^−1^ Hoechst 33342 (BD Biosciences) for 30 min at room temperature. After washing in PBS, the slides were mounted with Vectashield, and the images were collected using a CoolSNAP ES Olympus BX51 camera with the associated Metaview Software (Tokyo, Japan).

### ELISA assays

TCA8113 cells (1 × 10^5^ ml^−1^) were cultured for 48 h, and cell-free supernatants were collected and stored at −80 °C. Vascular endothelial growth factor (VEGF) concentrations were measured using the commercially available Human VEGF Quantikine ELISA Kit (R&D Systems, Minneapolis, MN, USA). The absorbance at 450 nm was determined using a microplate reader.

### Statistical analyses

All experiments were performed at least six times, and no blinding was performed. Values are presented as the mean±s.d. The statistical analyses were performed using two-tailed Student's *t*-test or analysis of variance with the SPSS 18.0 software (Chicago, IL, USA). *P*<0.05 was considered to indicate a statistically significant difference.

## Results

### ANG2 was upregulated in OSCC tissues

**Figure 1:**
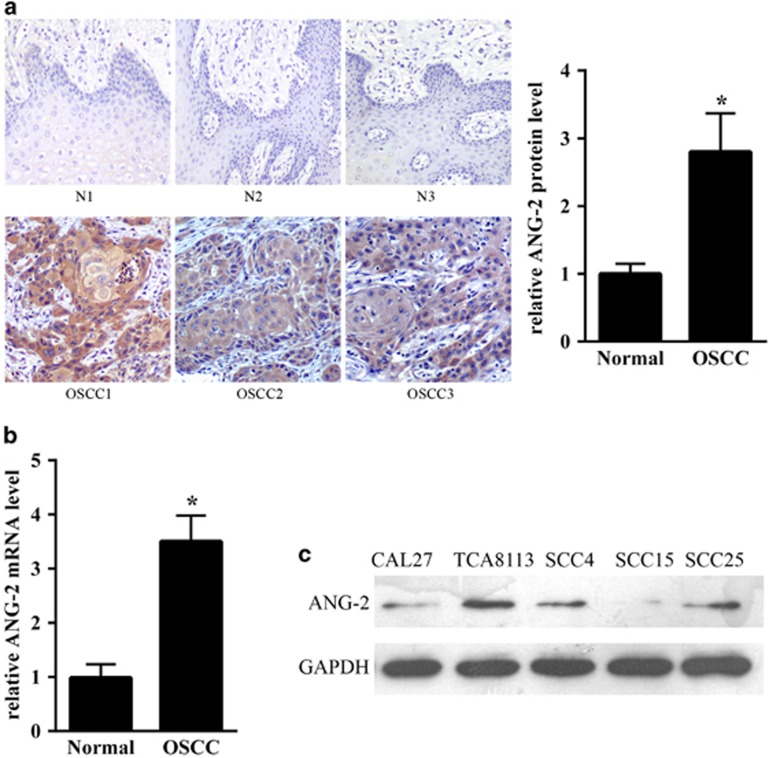
Angiopoietin 2 (ANG2) was upregulated in oral squamous cell carcinoma (OSCC) tissues. (**a**) Immunohistochemical and quantification analysis of ANG2 protein expression in normal liver tissue (up) and OSCC (bottom) samples using specific anti-ANG2 antibody. Representative photographs were obtained at × 200 magnification. (**b**) ANG2 mRNA expression levels in OSCC tissue samples and normal tissue samples (12 cases) were examined by real-time quantitative reverse transcriptase (qRT-PCR), respectively. (**c**) The expression of ANG2 in OSSC cell lines (TCA8113, cal27, SCC4, SCC15 and SCC25). GAPDH was used as an internal quantitative control. Three independent experiments were performed, and the data represent the means±s.d. *P<0.05.

To explorer the relationship between ANG2 and OSCC, OSCC tissue samples from 12 patients were examined by IHC analysis using an ANG2-specific antibody. The paired adjacent normal tissue samples were used as controls. ANG2 protein expression was increased in tumor tissues in comparison with normal tissue. Representative IHC and quantification analysis results from three patients are shown in Figure 1a. In addition, the level of ANG2 mRNA accumulation was also examined by real-time qRT-PCR using ANG2-specific primers. As shown in Figure 1b, ANG2 mRNA in OSCC tissues increased more than 3.5-fold compared with that in normal tissues. Taken together, these results indicated that ANG2 was significantly upregulated in OSCC tissues. We analyzed the expression of ANG2 in SCC cell lines (TCA8113, cal27, SCC4, SCC15 and SCC25), and TCA8113 cells were selected for further analyses because they expressed a significantly higher protein level of ANG2 compared with all other cell lines (Figure 1c).

### Effects of ANG2 on proliferation, apoptosis and the cell cycle in OSCC cells

**Figure 2:**
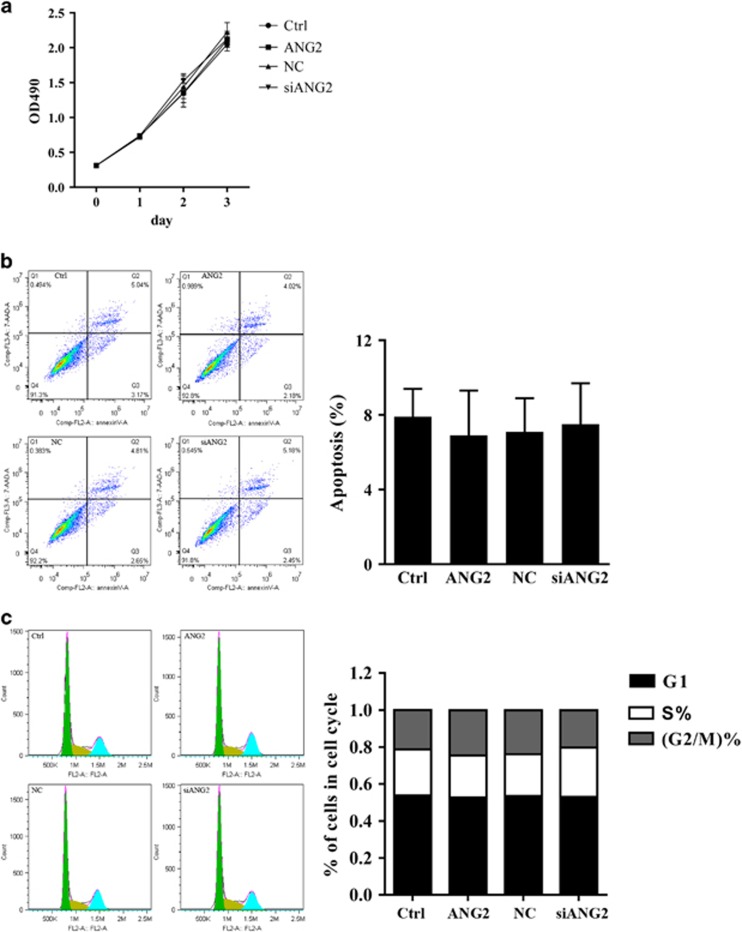
Effects of angiopoietin 2 (ANG2) on apoptosis and the cell cycle in oral squamous cell carcinoma (OSCC) cells. (**a**) Proliferation rates were determined by the MTS (3-(4, 5-dimethylthiazol-2-yl)-5-(3-carboxymethoxyphenyl)-2-(4-sulfophenyl)-2H-tetrazolium) assay on days 1–3 in TCA8113 mock cells or cells transfected with ANG2-encoded plasmids, ANG-targeted short interfering RNA (siRNA) or nonspecific (NC) siRNA. Before performing the MTS assays, cells were transfected with the indicated plasmids or siRNA for 24 h. Three independent experiments were performed, and the data represent the means±s.d. Treated and control groups of TCA8113 cells were used in subsequent experiments. (**b**) Representative dot plots showing fluorescence channel analysis of the treated and control groups of TCA8113 cells after dual staining with Annexin V and propidium iodide (PI) and analysis by flow cytometry. Columns represent the mean of three individual experiments; Bars, s.d. (**c**) Cell cycle distributions of the treated and control groups of TCA8113 cells were analyzed by flow cytometry. Data represent the mean±s.d. of three independent experiments.

We sought to explore the effect of ANG2 on OSCC cell proliferation. Plasmids encoding ANG2 mRNA were used to provide additional ANG2 expression in the OSCC cell line TCA8113. Moreover, the commercial siRNA targeting ANG2 mRNA sequences was used to inhibit ANG2 expression in TCA8113 cells. The nonspecific (NC) siRNA was used as a negative control. Briefly, TCA8113 cells were transfected with ANG2-encoded plasmid or ANG2-targeted siRNA. MTS assays were then performed to examine TCA8113 cell proliferation at different time points (Day 1, Day 2 and Day 3) 24 h after transfection. Mock cells (Ctrl) and cells transfected with NC siRNA served as controls. Our findings showed that either overexpression or inhibition of ANG2 had no effect on cell proliferation (Figure 2a).

To validate the cell proliferation results, Annexin V/propidium iodide-based apoptosis and flow cytometric analyses were performed to examine the effects of ANG2 on cell apoptosis and the cell cycle, respectively. As shown in Figure 2b, increasing or decreasing ANG2 expression did not affect apoptotic cell population in TCA8113 cells. Similarly, regulating the expression of ANG2 had no effect on the cell cycle distribution in TCA8113 cells (Figure 2c).

### Overexpression of ANG2 increased the migration and invasion of TCA8113 cells by regulating EMT

**Figure 3:**
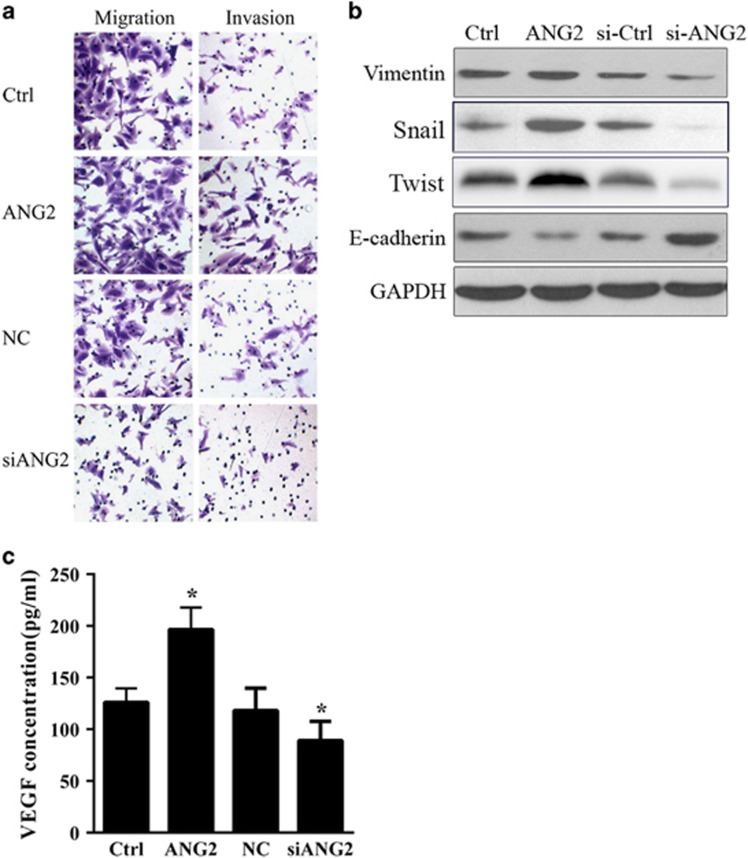
Overexpression of angiopoietin 2 (ANG2) increased the migration and invasion of TCA8113 cells by regulating epithelial–mesenchymal transition (EMT). (**a**) Cell migration and invasion were observed using a transwell chamber. (**b**) Western blot analysis of the cell proliferation-related proteins vimentin, Snail, Twist and E-cadherin in treated and control groups of TCA8113 cells. GAPDH served as a loading control. (**c**) Enzyme-linked immunosorbent assay (ELISA) was performed to analyze VEGF concentrations in cell-free supernatants. *Compared to Crtl or NC group. VEGF, vascular endothelial growth factor.

We sought to examine the role of ANG2 in the migration and invasion of TCA8113 cells. To achieve this goal, transwell migration and invasion assays were performed. As shown in Figure 3a, overexpression of ANG2 by transfecting ANG2-encoded plasmid increased the migration and invasion activities of TCA8113 cells, whereas inhibition of ANG2 by transfecting ANG2-targeted siRNA decreased TCA8113 cell migration and invasion.

EMT is a process by which epithelial cells lose their polarity and cell–cell adhesion and gain migratory and invasive properties, which has an important role in the initiation of tumor metastasis.^2^ Therefore, we sought to examine whether overexpression of ANG2 could affect the expressions of markers of EMT, such as vimentin, Snail, Twist and E-cadherin.^12, 13^ As shown in Figure 3b, the group of TCA8113 cells overexpressing ANG2 resulted in increased expression of vimentin, Snail and Twist, and reduced expression of E-cadherin, as compared with the group of TCA8113 mock cells. However, inhibition of ANG2 by ANG2-targeted siRNA transfection reduced the expression of vimentin, Snail and Twist proteins and increased the expression of E-cadherin protein, as compared with that in TCA8113 cells transfected with NC siRNA. A previous study showed that the downregulation of vimentin, Snail and Twist and the upregulation of E-cadherin could be an indicator of EMT.^14, 15, 16^ Therefore, our findings suggested that overexpression of ANG2 might increase cell migration and invasion by regulating the expression of vimentin, Snail, Twist and E-cadherin, and in turn affecting the EMT of OSCC cells.

As an important marker of tumor angiogenesis, VEGF has an important role in tumor growth and migration. In the present study, enzyme-linked immunosorbent assay (ELISA) was performed to analyze the VEGF concentrations in cell-free supernatants. The expression levels of VEGF in TCA8113 cells increased in response to ANG2 overexpression but decreased in response to ANG2 inhibition (Figure 3c).

### Overexpression of ANG2 promoted tumorigenicity, increased angiogenesis and reduced cell apoptosis in nude mice

**Figure 4:**
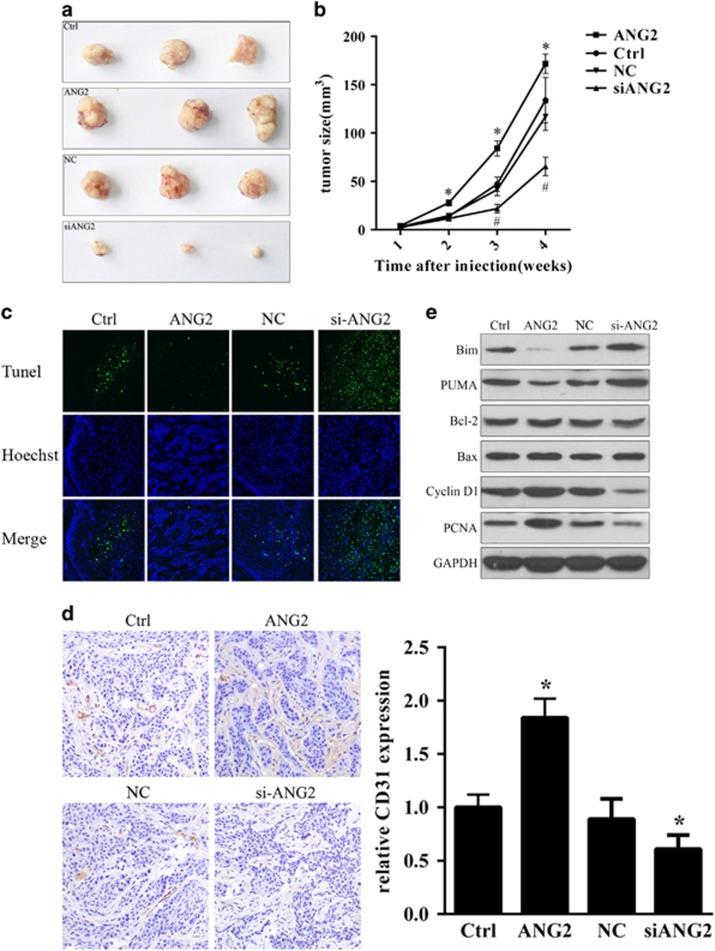
Overexpression of angiopoietin 2 (ANG2) promoted tumorigenicity, increased angiogenesis and reduced apoptosis in nude mice. Subcutaneous injection of treated and control groups of TCA8113 cells into nude mice. (**a**, **b**) Tumor volume was examined every week. Three independent experiments were performed, and the data represent the means±s.d. *P<0.05. (**c**) Tumor tissues from nude mice 4 weeks after injection were subjected to terminal deoxynucleotidyl transferase dUTP nick-end labeling (TUNEL) assays. Green TUNEL-positive cells are apoptotic cells with fragmented DNA, whereas blue Hoechst-positive cells represent all cells in this assay. Magnification is × 200. (**d**) Immunohistochemical and quantification analysis of CD31 protein expression in the treated and control groups of oral squamous cell carcinoma (OSCC) tissues with specific anti-CD31 antibody. Representative photographs were obtained at × 200 magnification. (**e**) Western blot analysis of tumor formation-related proteins including Bim, PUMA, Bcl-2, Bax, Cyclin D1 and PCNA in the treated and control groups of OSCC tissue samples. GAPDH served as a loading control.

After demonstrating that ANG2 has an important role in the migration and invasion of OSCC cells, we sought to examine tumor metastases *in vivo*. To achieve this goal, TCA8113 mock cells, TCA8113 cells transfected with ANG2-encoded plasmid or TCA8113 cells transfected with ANG2-targeted siRNA or NC siRNA were injected intravenously into nude mice, and the tumor volume was examined every week. As shown in Figure 4a and b, compared with the tumor groups induced by TCA8113 mock cells and NC siRNA-transfected cells, the ANG2-overexpressing cells exhibited a significant increase in tumor volume (*P*<0.05), whereas a significantly reduced tumor volume was observed in the groups in which ANG2 was inhibited (*P*<0.05), revealing a positive correlation between ANG2 and the formation of tumor tissues.

The tumor tissues from the treated and control groups were analyzed 4 weeks after injection by TUNEL assays. Our results showed that overexpression of ANG2 resulted in a reduced proportion of apoptotic cells, whereas inhibition of ANG2 increased the size of the apoptotic cell population (Figure 4c). These results suggested that overexpression of ANG2 might increase tumor tissue formation by reducing apoptosis. Interestingly, in our *in vitro* studies, increasing or decreasing the expression level of ANG2 did not affect cell growth or apoptosis (Figure 2), whereas the regulation of ANG2 expression *in vivo* resulted in significant changes in the growth of tumor tissues (Figure 4a and b), suggesting that the regulatory effects of ANG2 on tumor cells may depend on the *in vivo* microenvironment.

Given that overexpression of ANG2 could increase tumor growth and reduce cell apoptosis, we sought to identify whether ANG2 could affect the process of angiogenesis. Thus, IHC and quantification analyses were performed to examine the effect of ANG2 overexpression on CD31, a vascular marker that has an important role in angiogenesis.^17^ The results revealed increased expression of CD31 in tumor tissue groups of ANG2-overexpressing cells compared with the control groups, whereas reduced CD31 protein expression was observed in the groups with ANG2 inhibition (Figure 4d). These results indicated that overexpression of ANG2 could upregulate the protein expression of CD31 and thus facilitate angiogenesis.

To further explore the molecular basis of the ANG2 overexpression-induced increase in tumor growth and reduced apoptosis in tumor tissues, we examined tumor tissue proteins involved in tumor development by western blot analysis. First, we investigated apoptosis-related protein expression, including the pro-apoptotic Bcl-2 family (Bim, PUMA and BAX) and the anti-apoptotic Bcl-2 family (Bcl-2).^18, 19, 20^ Our results showed that overexpression of ANG2 reduced the protein expression of Bim and PUMA, whereas inhibition of ANG2 increased the protein expression of Bim and PUMA (Figure 4e). Moreover, neither increased nor decreased ANG2 expression affected the levels of Bcl-2 and Bax. These results suggested that overexpression of ANG2 may reduce cell apoptosis in tumor tissues by decreasing pro-apoptotic protein expression levels. Furthermore, we examined the cell proliferation-related proteins Cyclin D1 and PCNA, which are necessary for DNA replication and regulate G1/S phase transition.^21, 22^ As shown in Figure 4e, overexpression of ANG2 resulted in an increase in Cyclin D1 and PCNA expression, whereas inhibition of ANG2 reduced the expression of Cyclin D1 and PCNA, revealing a positive correlation between ANG2 and cell proliferation in tumor tissues.

## Discussion

ANG2 is thought to have an important role in angiogenesis.^5^ Numerous studies have demonstrated that tumor aggression could be the result of increased angiogenesis mediated by ANG2.^8, 9, 10^ The present study showed that overexpression of ANG2 increased OSCC tissue growth in nude mice. Moreover, overexpression of ANG2 resulted in the upregulation of CD31, which is a platelet endothelial cell adhesion molecule, an angiogenesis marker and functions as a bridge that links tumor cells and vascular endothelial cells and promotes the metastasis of malignant tumors.^17^ These results indicated that ANG2-induced abnormal angiogenesis promoted tumor progression.

Angiogenesis commonly requires EMT, one of the key processes underlying the acquisition of a migratory capacity by primary tumor cells.^2, 3, 4, 5^ E-cadherin, Snail, Twist and vimentin function as the key factors in the regulation of epithelial cell-to-cell adhesion.^15^ E-cadherin, an important glycoprotein of the classical cadherin members, regulates cell adhesion in normal adult epithelial cells.^12^ Loss of E-cadherin expression is considered to promote tumor invasion and metastasis, which can function as a marker of EMT.^4^ However, vimentin, a component of intermediate filaments, functions as a positive regulatory marker of EMT by upregulating several EMT-related genes.^13^ Snail and Twist also function as important EMT makers, and their abnormal expression can affect the EMT process, ultimately increasing the migration and invasion of tumor cells.^14, 16^ The positive correlation between ANG2 and VEGF indicated that angiogenesis had an important role in the progression of OSCC. We propose that ANG2 can induce abnormal EMT by regulating E-cadherin, Snail, Twist and vimentin, and affect VEGF expression, thus resulting in angiogenesis and tumor metastasis.

Beyond the induction of abnormal EMT, ANG2 could also induce increased angiogenesis by reducing cell apoptosis in OSCC tissues, which results from downregulation of the pro-apoptotic proteins Bim and PUMA. Apoptosis is commonly regulated by the mitochondrial apoptotic pathway, which is modulated by Bcl-2 family proteins possessing either pro- or anti-apoptotic activities and regulating the mitochondrial apoptotic pathway by controlling permeabilization of the outer mitochondrial membrane.^18^ The activities of anti-apoptotic proteins, such as Bcl-2 and Bcl-XL, protect mitochondrial integrity, whereas activation of pro-apoptotic proteins promotes the release of mitochondrial proteins such as cytochrome *c* and second mitochondria-derived activator of caspases into the cytosol, ultimately leading to cell death.^18^

In addition, dysregulation of the G1 to S transition is one of the most important reasons for tumor formation, which is tightly linked to unregulated PCNA and Cyclin D1 expression. PCNA and cyclin D1 are important mediators of the G1/S transition.^21, 22^ PCNA, a ring-shaped homotrimer clamp that loads on chromatin in an ATP-dependent manner, provides a sliding platform for various proteins involved in the DNA replication processes.^21^ Cyclin D1, together with CDK4/6 and activating CDK4/6, is essential for the transition from G0 into G1, preparing cells for the G1/S transition.^22^ The study presented herein showed that overexpression of ANG2 resulted in increased PCNA and cyclin D1 expression. Thus, abnormal expression of PCNA and cyclin D1 could induce a dysfunctional cell cycle and, in turn, promote angiogenesis and tumor growth.

Of note, our *in vitro* findings showed that ANG2 did not affect apoptosis or the cell cycle. In contrast, in the *in vivo* system, overexpression of ANG2 increased tumor growth. A previous study showed that differences in the effects induced by the regulation of ANG2 expression may demand the contributions of both endothelial cells and pericytes *in vivo* but not *in vitro*, where pericytes are absent.^23^ Our findings further support the notion that the *in vivo* microenvironment is required for the stimulatory effects of ANG2 on angiogenesis and OSCC tissue formation.

## Conclusion

In summary, the present findings showed that ANG2 was remarkably upregulated in OSCC tissues. Overexpression of ANG2 increased the migration and invasion of the OSCC cell line. Further investigations revealed that overexpression of ANG2 promoted tumor growth in nude mice. This stimulatory effect could be achieved by promoting angiogenesis mediated by the induction of abnormal EMT, as well as reducing apoptosis and increasing proliferation. These results indicate that ANG2 has an important role in the development of OSCC. Therefore, a specific enzymatic inhibitor of ANG2 may have therapeutic potential for the treatment of OSCC.

## Notes

This study was supported by Project of Science and Technology Department of Sichuan Province (No: 2014TD0011) and Project of Science and Technology Bureau of Chengdu (No: 2014-HM01-00415-SF). The authors declare no conflict of interest.
